# A surrogate reporter system for multiplexable evaluation of CRISPR/Cas9 in targeted mutagenesis

**DOI:** 10.1038/s41598-018-19317-x

**Published:** 2018-01-18

**Authors:** Hongmin Zhang, Yuexin Zhou, Yinan Wang, Yige Zhao, Yeting Qiu, Xinyi Zhang, Di Yue, Zhuo Zhou, Wensheng Wei

**Affiliations:** 1 Beijing Advanced Innovation Center for Genomics, Biodynamic Optical Imaging Center (BIOPIC), Peking-Tsinghua Center for Life Sciences, State Key Laboratory of Protein and Plant Gene Research, School of Life Sciences, Peking University, Beijing, 100871 China; 2 Academy for Advanced Interdisciplinary Studies, Peking University, Beijing, 100871 China

## Abstract

Engineered nucleases in genome editing manifest diverse efficiencies at different targeted loci. There is therefore a constant need to evaluate the mutation rates at given loci. T7 endonuclease 1 (T7E1) and Surveyor mismatch cleavage assays are the most widely used methods, but they are labour and time consuming, especially when one must address multiple samples in parallel. Here, we report a surrogate system, called UDAR (Universal Donor As Reporter), to evaluate the efficiency of CRISPR/Cas9 in targeted mutagenesis. Based on the non-homologous end-joining (NHEJ)-mediated knock-in strategy, the UDAR-based assay allows us to rapidly evaluate the targeting efficiencies of sgRNAs. With one-step transfection and fluorescence-activated cell sorting (FACS) analysis, the UDAR assay can be completed on a large scale within three days. For detecting mutations generated by the CRISPR/Cas9 system, a significant positive correlation was observed between the results from the UDAR and T7E1 assays. Consistently, the UDAR assay could quantitatively assess bleomycin- or ICRF193-induced double-strand breaks (DSBs), which suggests that this novel strategy is broadly applicable to assessing the DSB-inducing capability of various agents. With the increasing impact of genome editing in biomedical studies, the UDAR method can significantly benefit the evaluation of targeted mutagenesis, especially for high-throughput purposes.

## Introduction

Recent years have witnessed many changes and developments in genome editing technologies. Zinc-finger nucleases^1^, TALENs (transcription activator-like effector nucleases)^2–4^, and the CRISPR/Cas system^5–8^ have been widely used in biological or biomedical studies. In particular, the CRISPR/Cas system has become popular among researchers working in a variety of fields. For efficient genome editing using the CRISPR/Cas system, sgRNAs with high efficiency and specificity are crucial prerequisites^9,10^. Although various *in silico* tools for improving sgRNA design have been published^10–13^, experimental assessment of sgRNA efficiency is still needed because the complexity of cellular environments, such as chromatin accessibility, could affect sgRNA targeting^14^. For the above reasons, different techniques have been developed to assess the sgRNA efficiency, such as utilising Sanger sequencing and next-generation sequencing to directly analyse the sgRNA-targeting loci^15,16^ or using the Surveyor nuclease assay or T7E1 assay to detect mutation(s) by cleaving heteroduplex DNA at mismatches^6,17^. Other methods include a library-on-library method across thousands of genomic loci^18^ and a functional screening approach through antibody staining or phenotype enrichment^19^. However, the performance of the most commonly used sequencing strategies and Surveyor nuclease assays are highly affected by PCR specificity and polymorphism rates in certain organisms^20,21^.

A variety of surrogate reporter systems have been recently developed to evaluate targeted mutagenesis. In an episomal surrogate system, a proposed target site was placed upstream of the EGFP reporter gene, and the efficiency of RNA-guided endonucleases (RGEN) was evaluated based on how effective they could correct the shifted reading frame^22^. Similar strategies have been reported by correlating targeted mutations with the expression of EGFP or antibiotic-resistance reporter genes^23–25^. However, these methods are inefficient for assessing sgRNAs at a large scale because it is both time and labour consuming to construct a specific reporter for each individual sgRNA.

Here, we developed a new system for speedy and multiplexable evaluation of CRISPR/Cas-mediated or drug-induced mutagenesis. Our method requires only one universal PCR fragment as a surrogate reporter to assess targeted mutagenesis at distinct loci, thus providing a novel system for the convenient assessment of mutagenesis, especially for large-scale purposes.

## Results

### Linear donor integration at the double-strand breaks induced by Cas9/sgRNA

**Figure 1:**
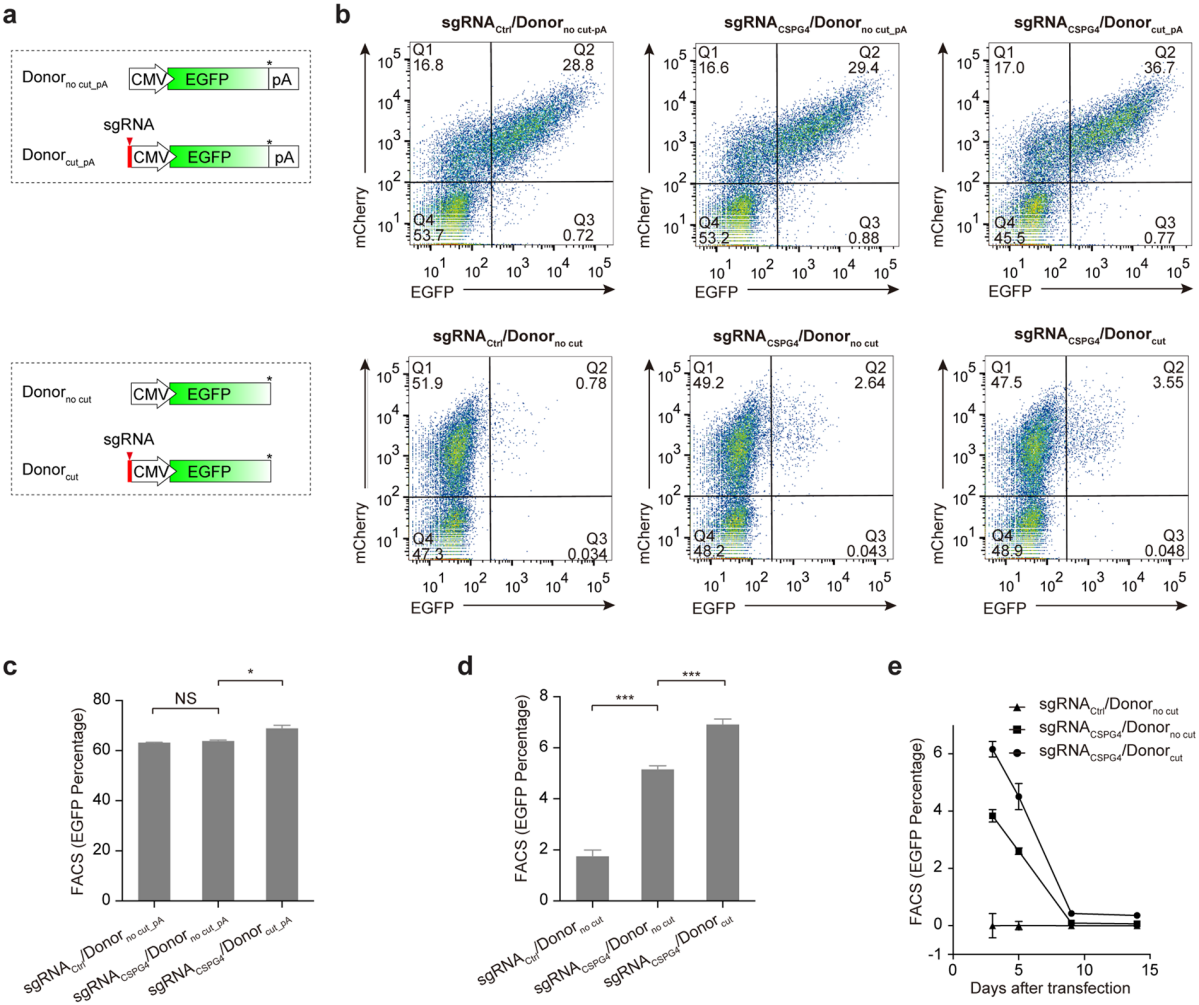
Establishment of a method for the evaluation of CRISPR/Cas-mediated targeted mutagenesis using a universal donor. (**a**) Schematic of linear donor fragment that contains a reporter system. The guide RNA cutting site is labelled at the 5′ end on the linear donors, as indicated. The stop codon is labelled with*. pA, polyA signal. (**b**) Representative flow cytometry plots of HeLa cells co-transfected with sgRNA and donors that consist of CMV-EGFP-pA (upper) or CMV-EGFP (lower) for three days. (**c**) FACS analysis for EGFP positivity in cells co-transfected with donors consisting of CMV-EGFP-pA and sgRNA/mCherry-expressing plasmids. The EGFP positivity has been normalised by mCherry expression using an equation of the form [Q2/(Q1 + Q2) × 100%]. Error bars indicate s.d. (n = 3), two-tailed p-values for the t-test. NS, not significant; *P < 0.05. (**d**) FACS analysis for EGFP positivity in cells co-transfected with donors consisting of CMV-EGFP and sgRNA/mCherry-expressing plasmids. Error bars indicate s.d. (n = 3), two-tailed p-values for the t-test. NS, not significant; ***P < 0.001. (**e**) FACS analysis for EGFP positivity in cells co-transfected with sgRNA and linear donors consisting of CMV-EGFP for the indicated number of days. The EGFP positivity was normalised by the transfection efficiency (mCherry), followed by subtracting EGFP positivity in the sgRNA_ctrl_ group. The error bars indicate s.d. (n = 3).

Our previous studies suggested that a linear donor fragment containing a reporter system can be integrated into the Cas9/sgRNA-targeting site^26^. We then questioned if we could apply this strategy to assess the sgRNA efficiency in targeted mutagenesis. To accomplish this goal, we test a linear donor-based system in the assessment of sgRNAs targeting the *CSPG4* gene. Two types of linear DNA donors were designed. One consists of a CMV-EGFP-polyA reporter cassette (Donor_no cut_pA_), and the other contains the same cassette with an sgRNA targeting site for *CSPG4* at its 5′ end (Donor_cut_pA_). For an experimental control, we removed the polyA tail from the aforementioned two types of donors and called them Donor_no cut_ and Donor_cut_ (Fig. 1a, lower). Next, a non-targeting sgRNA (sgRNA_Ctrl_) or an sgRNA targeting the *CSPG4* gene (sgRNA_CSPG4_) was co-transfected with donor fragments into HeLa cells that stably express Cas9^27^. After three days, cells were harvested and subjected to a fluorescence-activated cell sorting (FACS) assay. Upon co-transfection with polyA-containing donors (i.e., Donor_no cut_pA_ or Donor_cut_pA_), both the sgRNA_Ctrl_ and the sgRNA_CSPG4_ gave rise to substantial EGFP expression. Statistical analysis revealed that there was no significant difference in EGFP positivity among all groups (Fig. 1b, upper and Fig. 1c), which suggests that this strategy is infeasible for assessing targeted mutagenesis. In contrast, when co-transfection was performed with the donors without a polyA tail (i.e., Donor_no cut_ or Donor_cut_), the sgRNA_Ctrl_ produced low EGFP fluorescence signals, whereas the sgRNA_CSPG4_ produced much higher EGFP expression (Fig. 1b, lower and Fig. 1d). This finding suggested that when using polyA-free EGFP donors, the EGFP expression can specifically reflect sgRNA-targeted mutagenesis (see discussion). Next, we extended our observation for EGFP expression to two weeks and found EGFP signal peaks at day 3 post-co-transfection of sgRNA_CSPG4_ with EGFP donors (Fig. 1e). It is notable that when co-transfected with sgRNA_CSPG4_, EGFP donor fragments without an sgRNA targeting site (Donor_no cut_) successfully produced green fluorescence, albeit at a lower efficiency than Donor_cut_ (Fig. 1d,e). Using one universal donor without the sgRNA cutting site for the evaluation of mutagenesis at distinct loci will greatly simplify the experimental procedures, and therefore, we tested the universal Donor_no cut_ in the subsequent research. As such, taking advantage of the universal donor harbouring a CMV-EGFP reporter cassette free of polyA signal, we developed this novel surrogate system, which we designated UDAR (Universal Donor As Reporter), to assess targeted mutagenesis.

### UDAR assay is a reliable method for the evaluation of CRISPR/Cas-mediated targeted mutagenesis

**Figure 2:**
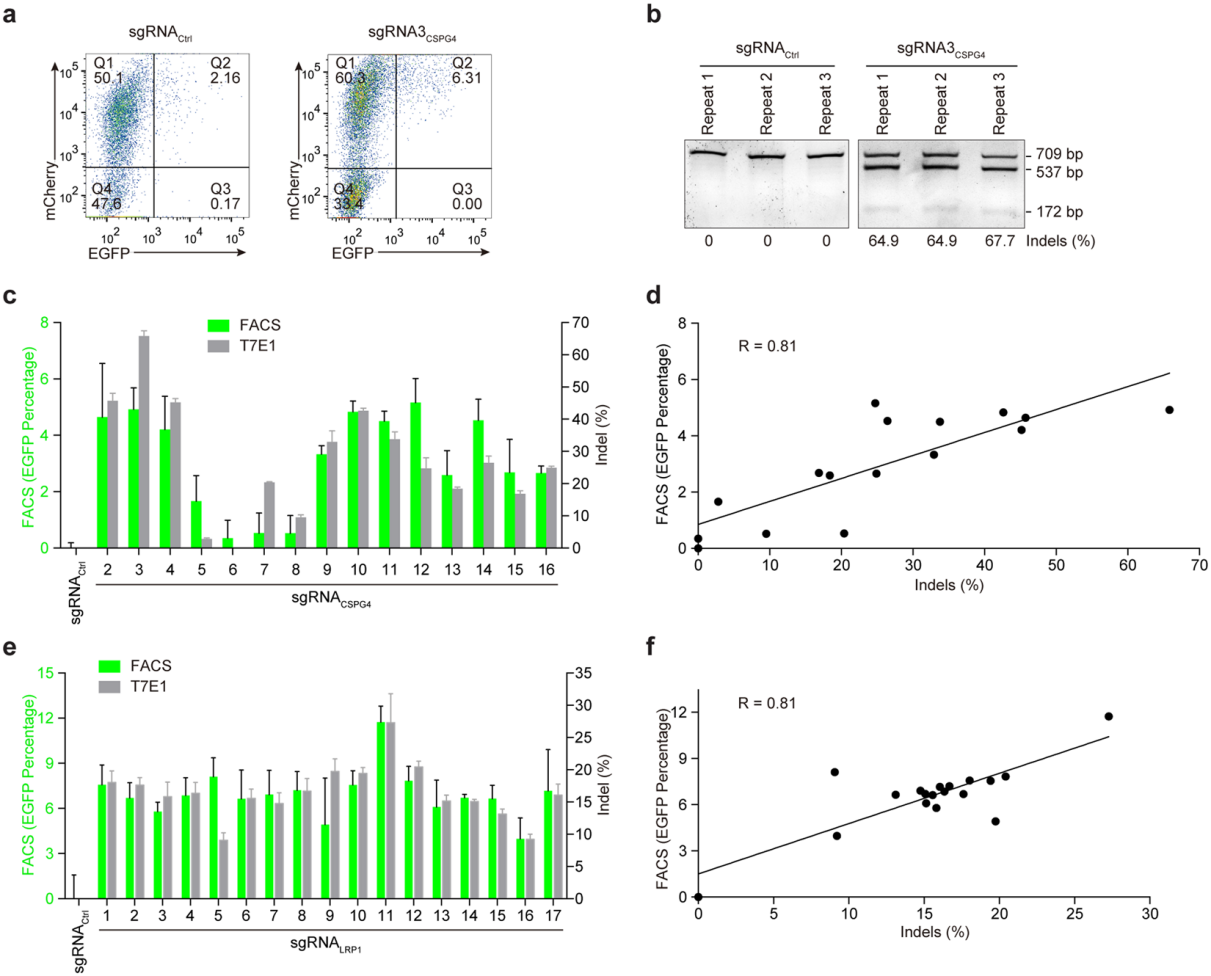
Assessment of the sgRNA efficiency by the UDAR assay in HeLa and HEK293T cells. (**a**) Representative flow cytometry plots of HeLa cells co-transfected with a universal donor and sgRNA. (**b**) Representative results of the T7E1 assay (three replicates are presented for each assay) at the *CSPG4* locus in the HeLa cells. Uncut (709 bp) and cut (537 bp and 172 bp) PCR bands are indicated. The indel ratios were calculated according to the band intensities. (**c**) Assessment of an sgRNA targeting the *CSPG4* gene by the UDAR and T7E1 assay in HeLa cells. EGFP percentages analysed by FACS (green) and indel ratios, measured by the T7E1 assay (grey), are plotted in the same graph. The error bars indicate s.d. (n = 3). (**d**) Correlation between EGFP percentages and indel ratios at the *CSPG4* locus in HeLa cells. Pearson’s correlation coefficient (R) = 0.81. (**e**) Assessment of an sgRNA targeting the *LRP1* gene by the UDAR and T7E1 assay in HEK293T cells. EGFP percentages analysed by FACS (green) and indel ratios, measured by the T7E1 assay (grey), are plotted in the same graph. The error bars indicate s.d. (n = 3). (**f**) Correlation between the EGFP percentages and indel ratios at the *LRP1* locus in the HEK293T cells. Pearson’s correlation coefficient (R) = 0.81.

To determine the reliability of our system based on universal donor integration, we compared the UDAR method with the T7E1 assay, one of the most widely used methods for mutagenesis detection. We randomly designed 15 sgRNAs that target distinct *CSPG4* loci and one non-targeting control sgRNA. The sgRNAs were then transfected alone or together with an EGFP donor into HeLa cells. After three days, cells co-transfected with sgRNA and donor were subjected to FACS analysis (Fig. 2a), and cells transfected with sgRNA alone were analysed by T7E1 assay (Fig. 2b). Compared with the non-targeting control group, all 15 sgRNA_CSPG4_ transfection groups showed significantly higher levels of EGFP expression to different degrees, which indicates that the targeting efficiency of sgRNA_CSPG4_ varies (Fig. 2c). Of note, the T7E1 assay also revealed varying sgRNA efficiency, with a similar pattern observed in FACS analysis (Fig. 2c). Statistical analysis demonstrated that the EGFP percentages from the UDAR method significantly correlated with the indel ratios detected by the T7E1 assay, demonstrating that our surrogate reporter faithfully reflects the sgRNA efficiency in the CRISPR/Cas9 system (Fig. 2d). Then, the efficacy of the UDAR assay at different gene loci and cell lines was further examined. We found that UDAR is also reliable for assessing sgRNAs targeting the *LRP1* gene (Fig. 2e,f and Supplementary Fig. S1) in HEK293T cells and the *CSPG4* gene (Supplementary Fig. S2) in HEK293T cells, which suggests that UDAR assay can be broadly applied.

**Figure 3:**
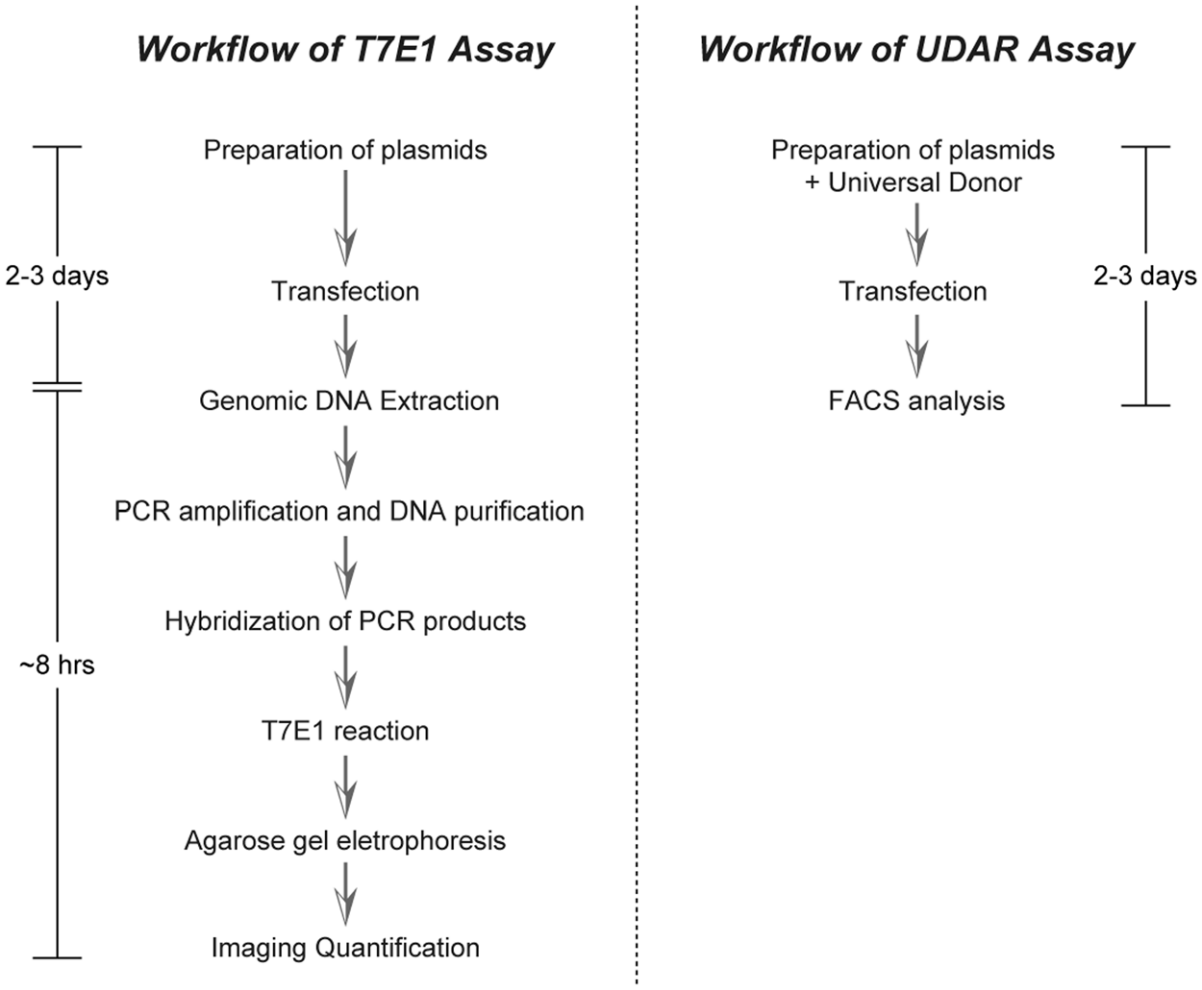
Workflow of the T7E1 and UDAR assays.

The experimental workflows for the T7E1 assay and UDAR assay are illustrated in Fig. 3. Compared with the T7E1 assay, which includes multiple hands-on processes, the UDAR assay is much simpler because after transfection, only a one-step FACS analysis is needed.

### UDAR assay is applicable for the detection of drug-induced double-strand breaks

**Figure 4:**
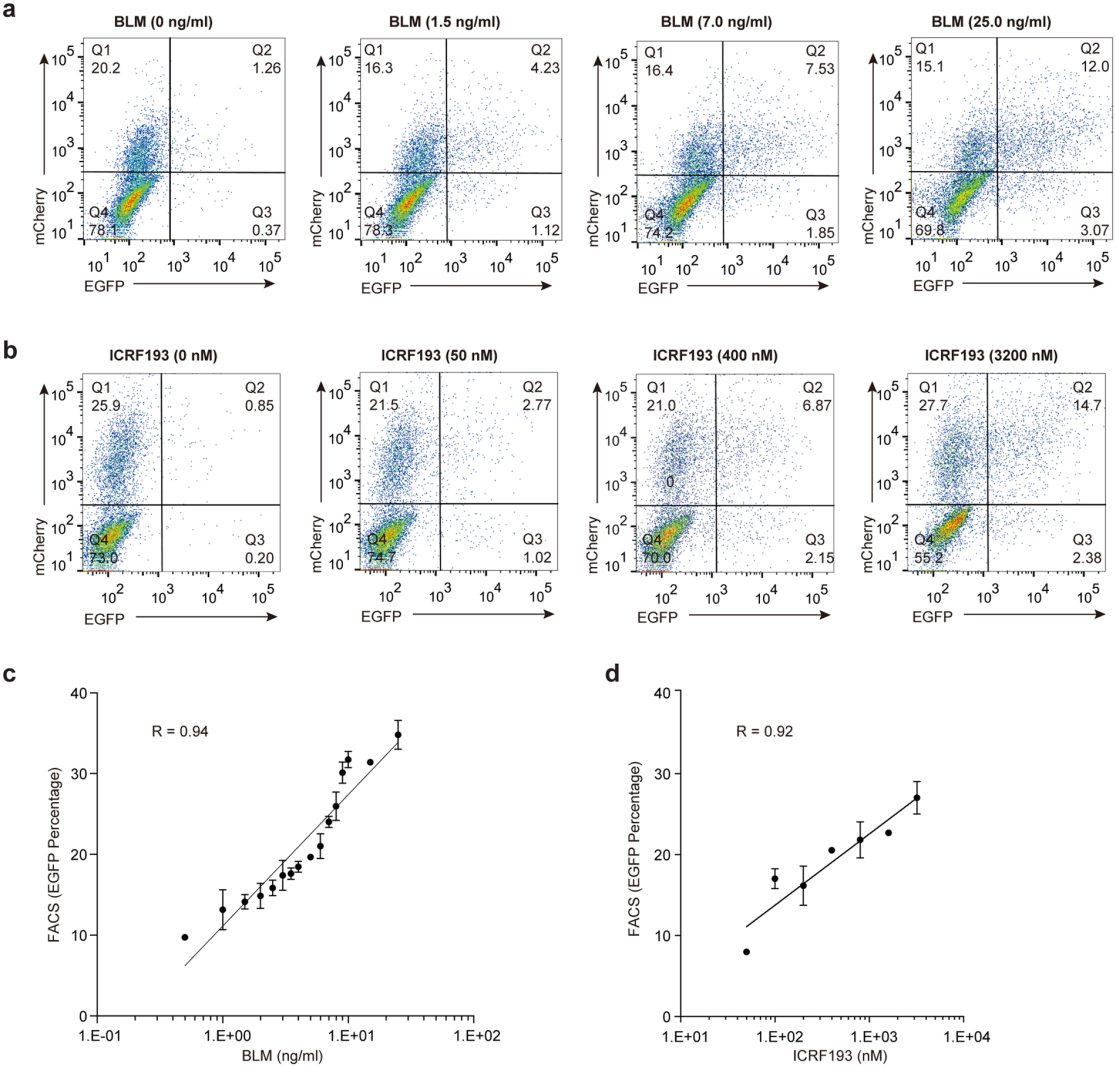
Analysis of drug-induced DSBs by the UDAR assay. (**a**,**b**) Universal Donor_no cut_ and mCherry-expressing plasmid (1 μg:0.1 μg) were co-transfected into HeLa cells in 6-well plates. Eight hours after transfection, the cells were exposed to different doses of bleomycin for 30 min (**a**) or ICRF193 for 24 h (**b**). After 48 h, the cells were subjected to FACS analysis. Representative flow cytometry plots are presented. (**c**,**d**) Correlation between EGFP percentages and doses of bleomycin (Spearman’s correlation coefficient (R) = 0.92) (**c**) or ICRF193 (Spearman’s correlation coefficient (R) = 0.92) (**d**). The error bars indicate s.d. (n = 3).

Because donor integration occurs at DSB sites, we inferred that the UDAR assay can be applied to evaluate the occurrence of DSBs induced by drugs. Thus, we utilised the UDAR assay to assess the DSBs caused by bleomycin, a radiomimetic drug that induces DSBs by free radical mechanisms^28^. We found that an increase in bleomycin resulted in an enhancement of the EGFP positive ratio (Fig. 4a). Moreover, a significant positive correlation was observed between the EGFP percentage and the bleomycin concentration (Spearman’s correlation coefficient: R = 0.94, Fig. 4c). Because the production of DSBs by bleomycin is concentration dependent^29,30^, it can be concluded that the UDAR assay can quantitatively assess the bleomycin-induced DSBs. Next, we tested if the UDAR assay is applicable for detecting the DSBs caused by ICRF193, a catalytic inhibitor of DNA topoisomerase II^31–33^. Similarly, the extent of EGFP expression correlates well with the ICRF193 concentration (Fig. 4b,d). Collectively, our results confirm that the UDAR assay is a powerful tool for assessing drug-induced DSBs, even for the large-scale screening of chemical compounds that induce double-strand breaks.

### UDAR assay provides an unbiased method for assessing sgRNA at a large scale

Our UDAR protocol requires a mere one-step co-transfection of the sgRNA/Cas9-expressing plasmid with the pre-made universal EGFP donor. Transfection can be performed in a multi-well format such that a large number of assays can be processed in parallel. Therefore, we are interested in testing whether our UDAR method is suitable for the library-scale assessment of sgRNA efficiency. We randomly designed 77 sgRNAs targeting the *ANTXR1* gene. In addition, we designed 18 non-targeting sgRNAs as negative controls. As such, a total of 95 sgRNAs comprised our large-scale experimental sets.

Using the UDAR approach, individual sgRNA together with the universal EGFP donor were transfected into HeLa cells in 24-well plates. After three days, we examined the EGFP positivity by using flow cytometry. Next, we compared the performance of UDAR with functional analysis, a method for sgRNA evaluations at a large scale^19^. *ANTXR1* encodes the cellular receptor of anthrax toxin, and disruption of this gene results in cellular resistance to the chimeric anthrax toxin PA/LFnDT^27,34^. We created a library containing all 95 sgRNAs that were delivered into HeLa cells by lentiviral infection. Two weeks after the infection, the cells were treated with PA/LFnDTA toxin for 48 h. The surviving cells were enriched after three rounds of PA/LFnDTA treatment, and the sgRNA-coding regions of both the pooled surviving cells and the toxin-untreated cells were analysed by deep sequencing analysis. In this functional assay, log2-fold changes, which represent the sgRNA enrichment level, were used to manifest the efficiency of the sgRNAs.

**Figure 5:**
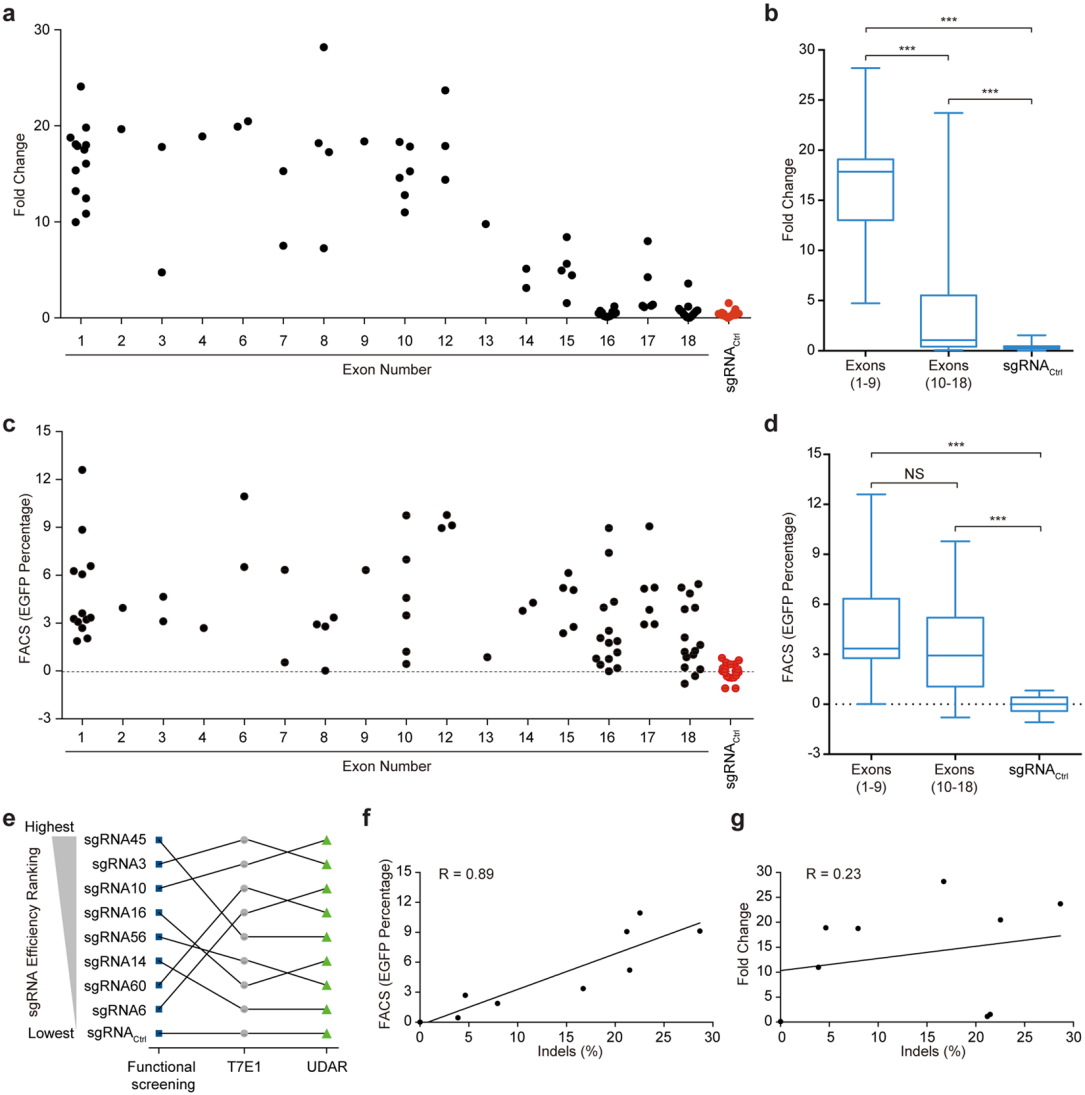
Library-scale evaluation of sgRNA efficiency. (**a**) Functional screening assay. Ninety-five sgRNAs were delivered into HeLa cells by lentiviral infection, followed by PA/LFnDTA toxin selection and deep sequencing for sgRNA-coding regions in the surviving cells for each of two replicates. Log2-fold changes represent the sgRNA enrichment level. The X axis indicates the sgRNA targeting location on the *ANTXR1* gene exons. (**b**) Statistical analysis for sgRNA activity in the first 9 exons (exons 1–9) and last 9 exons (exons 10–18) in a functional screening assay. Two-tailed p-values for the t-test. NS, not significant; ***P < 0.001. (**c**) UDAR assay for the evaluation of 95 sgRNAs. HeLa cells were transfected with individual sgRNA together with a universal EGFP donor for each of three replicates. After three days, the cells were subjected to FACS analysis. The performance of 77 sgRNAs specific for the *ANTXR1* gene (black) and 18 non-targeting control sgRNAs (red) are represented. (**d**) Statistical analysis for sgRNA activity in the first 9 exons (exons 1–9) and last 9 exons (exons 10–18) in the UDAR assay. Two-tailed p-values for the t-test. NS, not significant; ***P < 0.001. (**e**) Comparisons of the sgRNA efficiency ranking using the functional screening assay (blue), T7E1 assay (grey) and UDAR assay (green). (**f**) Correlation between indel ratios (T7E1) and EGFP ratios (UDAR). Pearson’s correlation coefficient (R) = 0.89. (**g**) Correlation between indel ratios (T7E1) and sgRNA enrichment fold (functional screening assay). Pearson’s correlation coefficient (R) = 0.23.

We found that the functional screening assay revealed that different sgRNAs have varying activities from exon 1 to exon 18 (Fig. 5a). Statistical analysis indicated that the efficiency of the sgRNAs that target the first 9 exons (exons 1–9) is significantly higher than the efficiency of the sgRNAs that target the last 9 exons (exons 10–18) based on the functional assay (Fig. 5b). This phenomenon likely occurs because frameshift mutations close to the 3′ end of a gene are less likely to disrupt the gene function^19^. Notably, sgRNAs that target the exons toward the 3′ end of the gene (exon 13–18) appeared to be more effective according to the UDAR assay than according to functional evaluation (Fig. 5c). In addition, no position-related bias was observed in the UDAR assay (Fig. 5d).

To determine which method is more reliable in large-scale sgRNA assessment, we compared the results from the UDAR or functional assay with those from the T7E1 assay. We chose 6 sgRNAs (sgRNA6, 14, 16, 45, 56, and 60) that show distinct activity between the UDAR and functional assays. Moreover, 2 sgRNAs showed similar results in both assays (sgRNA 3 and 10), and 1 non-targeting sgRNA (sgRNA_Ctrl_) was also included (Supplementary Fig. S3). Upon pooling the data together, the UDAR method showed a much higher level of consistency with the T7E1 assay compared to the functional analysis (Fig. 5e). Indeed, statistical analysis demonstrated that the sgRNA activity obtained from the UDAR assay significantly correlated with the activity observed in the T7E1 assay (Fig. 5f), whereas the functional assay failed to exhibit such a correlation (Fig. 5g). Altogether, our data demonstrated that the UDAR assay is an unbiased method for the evaluation of targeted mutagenesis at a library scale.

## Discussion

In the current study, we have developed a novel method, UDAR, for convenient, reliable, and reproducible assessment of the sgRNA efficiency, especially for high-throughput purposes. The UDAR method has several advantages: (1) It is easy to use. UDAR requires one universal PCR fragment for distinct loci. (2) It is time and labour saving. Once a universal donor containing a reporter system has been prepared in advance, this method requires only a one-step transfection and FACS analysis with reduced hands-on time. (3) It can be adapted to high-throughput applications. Both transfection and FACS analysis can be performed in a high-throughput format such that this method provides the possibility of screening highly efficient sgRNAs in a genome-wide approach, especially when an automated liquid handling system is applied. (4) It is not affected by the sequence of the sgRNA target site. For PCR amplification in the T7E1 assay or other PCR-based methods, it is sometimes difficult to specifically amplify the target sequence in the genome DNA because of the lack of suitable primers. In addition, for organisms with a high rate of polymorphism in the genome, T7E1 or Surveyor assays could generate false positive results^35^. In contrast, the UDAR assay is independent of genome amplification procedures. (5) It is an unbiased method. Compared with the functional screening strategy, which is affected by the location of the sgRNA target site, the UDAR assay is based on DSB occurrences and makes an unbiased evaluation for all sgRNAs.

Because the UDAR assay is based on NHEJ-mediated donor integration, which is homology independent, EGFP donors can be integrated into spontaneous DSBs. Thus, compared to the T7E1 assay, which specifically detects the DNA mismatch at given loci, the UDAR method could more likely result in false positive results. However, in multiple experiments, we observed surprisingly high positive correlations between the UDAR and T7E1 results. Because the evaluation of all the sgRNAs, including non-targeting control sgRNAs, was performed in the same cell line in parallel, we reason that the normalisation of sgRNA values to the control group could minimise off-target effects that are caused by unintended DSBs.

Using the polyA-free EGFP donor is the key for the UDAR assay to work. It is well known that the polyA signal is vital for mRNA nuclear export, stability and translation^36,37^. Thus, in cells co-transfected with a non-targeting sgRNA, mRNA transcribed from the non-integrated polyA-free EGFP donor can be degraded rapidly, which results in a very low EGFP signal. However, once the polyA-free donor is integrated at the targeted loci, the polyA tail of the targeted gene could be transcribed following the stop codon of the CMV-EGFP cassette. This “gain of polyA” mechanism thus ensures EGFP mRNA stabilisation and expression in cells upon donor integration. In contrast, transfection of the EGFP donor harbouring a polyA tail could result in robust EGFP expression in spite of the donor integration, which makes it difficult to assess the targeted mutagenesis based on the EGFP expression. Notably, EGFP expression upon UDAR integration requires its 3′ polyA signal. The fact that the UDAR result correlated well with the T7E1 assay result suggests that UDAR integrations at the antisense orientation have little effect on the sgRNA effect.

It is noteworthy that the EGFP positive ratio decreased with prolonged transfection time. This “unstable integration” phenomenon was observed in a number of studies^38–40^. It has been proposed that the recipient genomic loci are often unstable after this non-homologous or “illegitimate” integration^41^, while the detailed mechanism by which expression of the integrated donor declines with time is not well understood. In our study, Donor_no cut_ combined with Cas9/sgRNA can be used for evaluating the sgRNA activity, while it is better to use Donor_cut_ to efficiently generate gene knock-in or create mutagenesis, which has been described in our previous study^26^.

Current anticancer treatments rely heavily on a combination of genotoxic agents, such as chemical compounds that induce DSBs, along with other cancer drugs^42,43^. Thus, assessing the efficiency of various DSB-generating drugs is of great significance. γH2AX foci counting is a method commonly used for this purpose, and it requires immunofluorescent staining with specific antibodies^44^. However, γH2AX foci counting is imprecise and time consuming because it requires human intervention for foci definition and manual adjustment^45^. Thus, the UDAR assay that we developed can significantly facilitate the evaluation of genotoxic agents.

Since the CRISPR/Cas system was successfully used to edit the human genome, a large amount of effort has been made to find sgRNAs with high efficiency and specificity. Our research provides a quick and reliable method to meet this urgent need. In combination with *in silico* design, the UDAR assay can help to determine the most effective sgRNAs, thus contributing to both optimising the sgRNA design criteria and improving the performance of genome-wide sgRNA libraries. Furthermore, the UDAR assay might be applicable in other contexts, such as gene tagging, live visualisation of genomic loci, or quantification of DSBs caused by drugs.

## Methods and Materials

### Cell cultures and transfection

HeLa cells that stably express Cas9 protein^27^ and HEK293T cells were maintained in Dulbecco’s modified Eagle’s medium (DMEM, 10–013-CV, Corning, Tewksbury, MA, USA) with 1% penicillin/streptomycin and 10% foetal bovine serum (FBS, Lanzhou Bailing Biotechnology Co., Ltd. Lanzhou, China). All the cells were maintained at 37 °C under 5% CO_2_. For transfection, 2 × 10^5^ HeLa cells or 4 × 10^5^ HEK293T cells were seeded on 6-well plates and transfected with X-tremeGENE HP (06366546001, Roche, Mannheim, German) according to the supplier’s protocols.

### Linear donor preparation

To serve as a universal template, donors containing the CMV-driven EGFP gene and protection sequences (listed in Supplementary Sequences) were pre-generated and cloned into the TA cloning vector (CT501–02, TransGen Biotech, Beijing, China). With primers (listed in Supplementary Table S1) specific for the template, we performed a one-step PCR reaction using the *Trans Taq*^®^ DNA Polymerase High Fidelity (HiFi) kit (K10222, TransGen Biotech, Beijing, China) according to the supplier’s protocols. Then, the PCR-amplified DNA was purified with the DNA Clean & Concentrator-25 kit (D4034, Zymo Research, Orange, CA, USA).

### gRNA-expressing plasmid cloning

sgRNA oligonucleotides were synthesised (Ruibiotech, Beijing) and cloned into a backbone vector with an mCherry-coding sequence as described elsewhere^26^, and the sgRNA-coding sequences are listed in the Supplementary data.

### UDAR assay for assessing the sgRNA efficiency

For the HeLa cells that stably express Cas9 protein, sgRNA and Donor_no cut_ (1 μg:1 μg) were co-transfected into cells in six-well plates. For the HEK293T cells, the Cas9-expressing plasmid, sgRNA, and Donor_no cut_ (0.9 μg:0.9 μg:0.2 μg) were co-transfected into cells in six-well plates. Forty-eight hours after transfection, the cells were analysed by FACS to determine their EGFP positivity. The EGFP positivity was normalised by the transfection efficiency determined by mCherry positivity, followed by subtracting EGFP positivity in the sgRNA_Ctrl_ control group.

### UDAR assay for assessing drug-induced DSBs

For the HeLa cells, universal Donor_no cut_ and mCherry-expressing plasmid (1 μg:0.1 μg) were co-transfected into 2 × 10^5^ cells pre-seeded in 6-well plates. Eight hours after transfection, the cells were exposed to different doses of bleomycin for 30 min or ICRF193 for 24 h. After 48 h, the cells were subjected to FACS analysis.

### T7E1 assay for assessing the sgRNA efficiency

sgRNA and a plasmid with puromycin resistance (0.5 μg:0.1 μg) were co-transfected into HeLa cells, followed by selection with 1 μg/ml puromycin two days later. The Cas9-expressing plasmid, sgRNA, and a plasmid with puromycin resistance (0.9 μg:0.9 μg:0.1 μg) were co-transfected into HEK293T cells, which were then selected with 2 μg/ml puromycin two days later. After collecting the puromycin-resistant cells, genomic DNA was prepared with the Dneasy Blood & Tissue Kit (69504, Qiagen, Hilden, Germany). For the PCR amplification of sgRNA-targeting genome regions with the corresponding primers (listed in Supplementary Table S2), we used the *Trans Taq* DNA Polymerase High Fidelity (HiFi) kit (K10222, TransGen Biotech, Beijing, China) according to the supplier’s protocols. Then, the purified PCR products were digested with 0.5 μl T7 nuclease (M0302L, NEB, Massachusetts, USA) in a 50-μl volume at 37 °C for 20 min.

### Functional screening for assessing the sgRNA efficiency

A library containing all the 95 sgRNAs was delivered into HeLa cells by lentiviral infection at an MOI of 0.3. After 48 h, EGFP positive cells were enriched by FACS analysis. For functional screening, the enriched cells were subjected to three rounds of PA/LFnDTA treatment (PA: 100 ng/ml; LFnDTA: 50 ng/ml) for each of two replicates. Then, the surviving cells, together with an original cell library (toxin untreated), were collected and subjected to deep-sequencing analysis for the sgRNA-coding regions. sgRNAs were ranked by the average log2-fold changes of the normalised counts. The primers used for PCR amplification of the sgRNA-coding regions are listed in Supplementary Table S3.

### Data availability

All the data generated or analysed during this study are included in this published article (and its Supplementary Information files).

## Electronic supplementary material

supplementary information
