# Supplementary material for: A surrogate reporter system for multiplexable evaluation of CRISPR/Cas9 in targeted mutagenesis
Source: Sci Rep. 2018 Jan 18;8:1042. doi: 10.1038/s41598-018-19317-x (PMC5773543; doi:10.1038/s41598-018-19317-x)
Supplement: Supplementary file 1 — supplementary information [file 41598_2018_19317_MOESM1_ESM.pdf]

A surrogate reporter system for multiplexable evaluation of CRISPR/Cas9 in targeted mutagenesis

Hongmin Zhang[1,2], Yuexin Zhou[1], Yinan Wang[1,2], Yige Zhao[1], Yeting Qiu[1], Xinyi Zhang[1], Di Yue[1], Zhuo Zhou[1,*] & Wensheng Wei[1,*]

[1]Beijing Advanced Innovation Center for Genomics, Biodynamic Optical Imaging Center (BIOPIC), Peking-Tsinghua Center for Life Sciences, State Key Laboratory of Protein and Plant Gene Research, School of Life Sciences, Peking University, Beijing 100871, China. [2]Academy for Advanced Interdisciplinary Studies, Peking University, Beijing 100871, China.

*Correspondence should be addressed to W.W. (wswei@pku.edu.cn) and Z.Z.(zhouzhuo@pku.edu.cn).

SUPPLEMENTARY SEQUENCES

EGFP donor (CMV-EGFP)

TCCACTGCGACGTCGCGAGTGGTATGCCCCTCGCTAATACTTAGTCACCTACTAGTTAGTCAATAGTAAT
CAATTACGGGGTCATTAGTTCATAGCCCATATATGGAGTTCCGCGTTACATAACTTACGGTAAATGGCCC
GCCTGGCTGACCGCCCAACGACCCCCGCCCATTGACGTCAATAATGACGTATGTTCCCATAGTAACGCC
AATAGGGACTTTCCATTGACGTCAATGGGTGGAGTATTTACGGTAAACTGCCCACTTGGCAGTACATCAA
GTGTATCATATGCCAAGTACGCCCCCTATTGACGTCAATGACGGTAAATGGCCCGCCTGGCATTATGCCC
AGTACATGACCTTATGGGACTTTCCTACTTGGCAGTACATCTACGTATTAGTCATCGCTATTACCATGGTG
ATGCGGTTTTGGCAGTACATCAATGGGCGTGGATAGCGGTTTGACTCACGGGGATTTCCAAGTCTCCAC
CCCATTGACGTCAATGGGAGTTTGTTTTGGCACCAAAATCAACGGGACTTTCCAAAATGTCGTAACAACT
CCGCCCCATTGACGCAAATGGGCGGTAGGCGTGTACGGTGGGAGGTCTATATAAGCAGAGCTGGTTTAG
TGAACCGTCAGATCCGCTAGCGCTACCGGTCGCCACCATGGTGAGCAAGGGCGAGGAGCTGTTCACCG
GGGTGGTGCCCATCCTGGTCGAGCTGGACGGCGACGTAAACGGCCACAAGTTCAGCGTGTCCGGCGAG
GGCGAGGGCGATGCCACCTACGGCAAGCTGACCCTGAAGTTCATCTGCACCACCGGCAAGCTGCCCGT
GCCCTGGCCCACCCTCGTGACCACCCTGACCTACGGCGTGCAGTGCTTCAGCCGCTACCCCGACCACA
TGAAGCAGCACGACTTCTTCAAGTCCGCCATGCCCGAAGGCTACGTCCAGGAGCGCACCATCTTCTTCA
AGGACGACGGCAACTACAAGACCCGCGCCGAGGTGAAGTTCGAGGGCGACACCCTGGTGAACCGCATC
GAGCTGAAGGGCATCGACTTCAAGGAGGACGGCAACATCCTGGGGCACAAGCTGGAGTACAACTACAA
CAGCCACAACGTCTATATCATGGCCGACAAGCAGAAGAACGGCATCAAGGTGAACTTCAAGATCCGCCA
CAACATCGAGGACGGCAGCGTGCAGCTCGCCGACCACTACCAGCAGAACACCCCCATCGGCGACGGCC
CCGTGCTGCTGCCCGACAACCACTACCTGAGCACCCAGTCCGCCCTGAGCAAAGACCCCAACGAGAAG
CGCGATCACATGGTCCTGCTGGAGTTCGTGACCGCCGCCGGGATCACTCTCGGCATGGACGAGCTGTA
CAAGTAGTAATTGATTAGTAGATAAGTGAGGGCGTAACAATCCTAAGCCCGCGATCTATAACAGATCGG

EGFP donor (CMV-EGFP-polyA)

TCCACTGCGACGTCGCGAGTGGTATGCCCCTCGCTAATACTTAGTCACCTACTAGTTAGTCAATAGTAAT
CAATTACGGGGTCATTAGTTCATAGCCCATATATGGAGTTCCGCGTTACATAACTTACGGTAAATGGCCC
GCCTGGCTGACCGCCCAACGACCCCCGCCCATTGACGTCAATAATGACGTATGTTCCCATAGTAACGCC
AATAGGGACTTTCCATTGACGTCAATGGGTGGAGTATTTACGGTAAACTGCCCACTTGGCAGTACATCAA
GTGTATCATATGCCAAGTACGCCCCCTATTGACGTCAATGACGGTAAATGGCCCGCCTGGCATTATGCCC
AGTACATGACCTTATGGGACTTTCCTACTTGGCAGTACATCTACGTATTAGTCATCGCTATTACCATGGTG
ATGCGGTTTTGGCAGTACATCAATGGGCGTGGATAGCGGTTTGACTCACGGGGATTTCCAAGTCTCCAC
CCCATTGACGTCAATGGGAGTTTGTTTTGGCACCAAAATCAACGGGACTTTCCAAAATGTCGTAACAACT
CCGCCCCATTGACGCAAATGGGCGGTAGGCGTGTACGGTGGGAGGTCTATATAAGCAGAGCTGGTTTAG
TGAACCGTCAGATCCGCTAGCGCTACCGGTCGCCACCATGGTGAGCAAGGGCGAGGAGCTGTTCACCG

GGGTGGTGCCCATCCTGGTCGAGCTGGACGGCGACGTAAACGGCCACAAGTTCAGCGTGTCCGGCGAG
GGCGAGGGCGATGCCACCTACGGCAAGCTGACCCTGAAGTTCATCTGCACCACCGGCAAGCTGCCCGT
GCCCTGGCCCACCCTCGTGACCACCCTGACCTACGGCGTGCAGTGCTTCAGCCGCTACCCCGACCACA
TGAAGCAGCACGACTTCTTCAAGTCCGCCATGCCCGAAGGCTACGTCCAGGAGCGCACCATCTTCTTCA
AGGACGACGGCAACTACAAGACCCGCGCCGAGGTGAAGTTCGAGGGCGACACCCTGGTGAACCGCATC
GAGCTGAAGGGCATCGACTTCAAGGAGGACGGCAACATCCTGGGGCACAAGCTGGAGTACAACTACAA
CAGCCACAACGTCTATATCATGGCCGACAAGCAGAAGAACGGCATCAAGGTGAACTTCAAGATCCGCCA
CAACATCGAGGACGGCAGCGTGCAGCTCGCCGACCACTACCAGCAGAACACCCCCATCGGCGACGGCC
CCGTGCTGCTGCCCGACAACCACTACCTGAGCACCCAGTCCGCCCTGAGCAAAGACCCCAACGAGAAG
CGCGATCACATGGTCCTGCTGGAGTTCGTGACCGCCGCCGGGATCACTCTCGGCATGGACGAGCTGTA
CAAGTAGTCCGGACTCAGATCTCGAGCTCAAGCTTCGAATTCTGCAGTCGACGGTACCGCGGGCCCGG
GATCCACCGGATCTAGATAACTGATCATAATCAGCCATACCACATTTGTAGAGGTTTTACTTGCTTTAAAA
AACCTCCCACACCTCCCCCTGAACCTGAAACATAAAATGAATGCAATTGTTGTTGTTAACTTGTTTATTGC
AGCTTATAATGGTTACAAATAAAGCAATAGCATCACAAATTTCACAAATAAAGCATTTTTTTCACTGCATTC
TAGTTGTGGTTTGTCCAAACTCATCAATGTATCTTATAATTGATTAGTAGATAAGTGAGGGCGTAACAATC
CTAAGCCCGCGATCTATAACAGATCGG

PCR template sequence for donor construction. CMV promoter region is in black, gene encoding EGFP is in green, SV40-polyA-terminator is in blue, stop codon(TAG) is shaded in light grey, protection sequences are shown in red.

SUPPLEMENTARY FIGURES

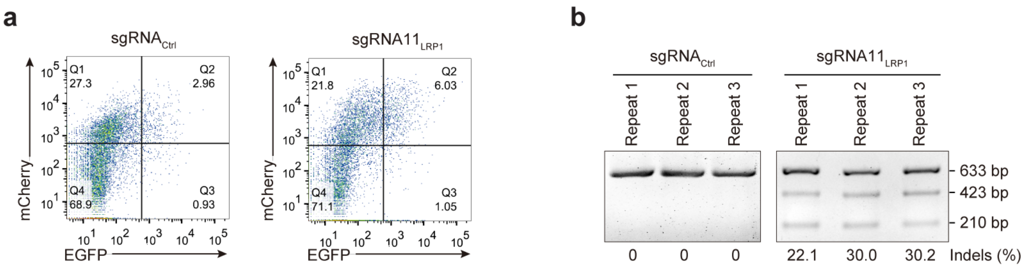

Supplementary Figure 1. Assessment of sgRNA efficiency by UDAR assay at *LRP1* locus in HEK293T cells. (**a**) Representative flow cytometry plots of HEK293T cells co-transfected with universal donor and sgRNA/Cas9. (**b**) Representative results of T7E1 assay (three replicates were presented for each assay) at the *LRP1* locus in HEK293T cells. Uncut (733 bp) and cut (423 bp and 210 bp) PCR bands are indicated. Indel ratios were calculated according to band intensities.

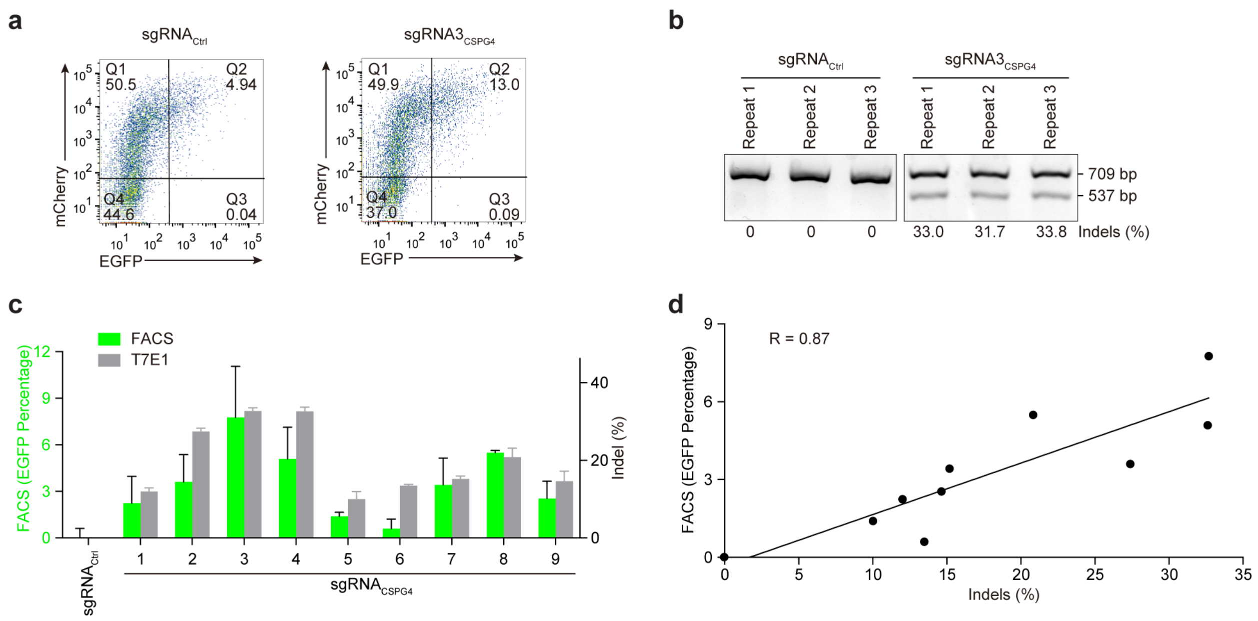

Supplementary Figure 2. Assessment of sgRNA efficiency by UDAR assay at *CSPG4* locus in HEK293T cells. (**a**) Representative flow cytometry plots of HEK293T cells co-transfected with universal donor and sgRNA/Cas9p. (**b**) Representative results of T7E1 assay (three replicates were presented for each assay). Uncut (709 bp) and cut (537 bp) PCR bands are indicated. Indel ratios were calculated according to band intensities. (**c**) In HEK293T cells, EGFP percentages analyzed by FACS (green) and indel ratios measured by T7EI assay (grey) were plotted in the same graph. Error bars indicate s.d. (n = 3). (**d**) Correlation between EGFP percentages and indel ratios at the *CSPG4* locus in HEK293T cells. Pearson's correlation coefficient (R) = 0.87.

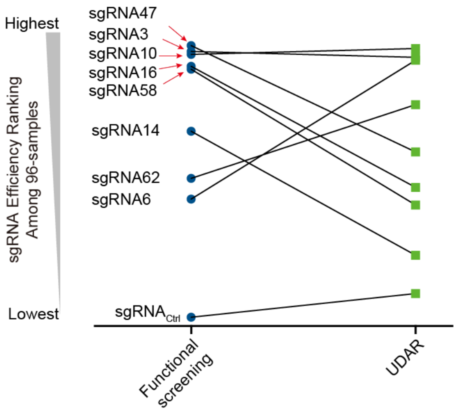

Supplementary Figure 3. sgRNAs selected for experiments in **Figure 5** (**e**), (**f**) and (**g**).

Figure 2b-left

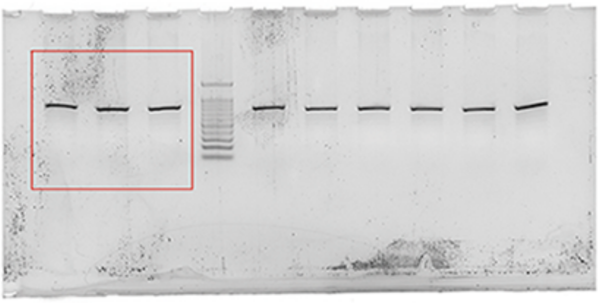

Figure 2b-right

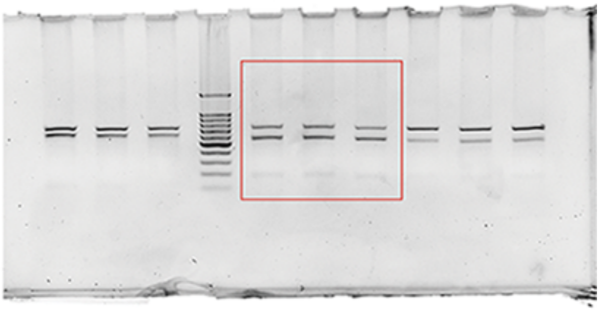

Supplementary Figure 1b-left

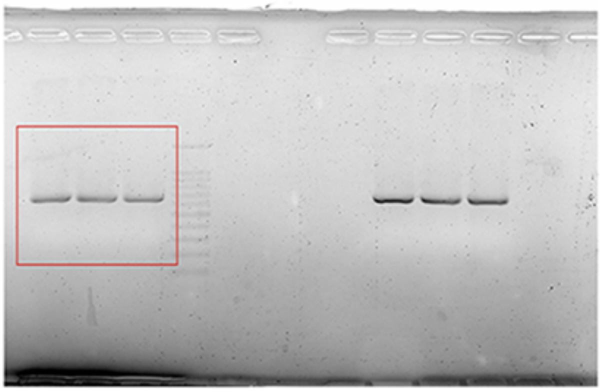

Supplementary Figure 1b-right

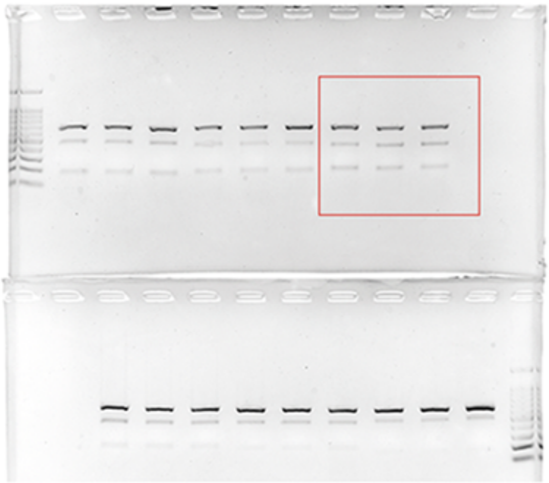

Supplementary Figure 2b-left

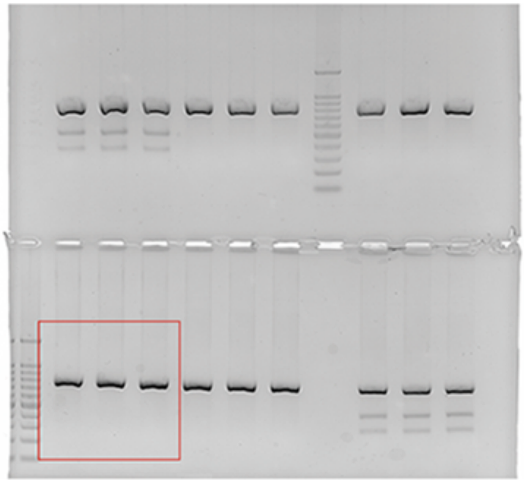

Supplementary Figure 2b-right

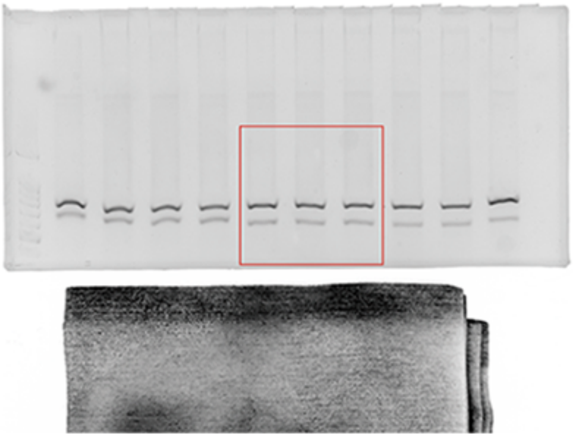

Supplementary Figure 4. Gels used in the figures.

SUPPLEMENTARY TABLES

Supplementary Table 1. PCR primers for linear donor construction.

Donor	Primers (Forward/Reverse)	
	Step 1	Step 2
$Donor_{cut}$ (CMV-EGFP / CMV-EGFP-polyA)	5'-AGCTCCCAGGACTCCGCTGGCTCCTCGC AATAGTAATCAATTACGGGGTCATTAGTTC-3' /5'-GGCTTAGGATTGTTAC GCCCTCACTTATCTACTAATCAATTA-3'	5'-TCCACTGCGACGTCGCGAGTCCACTCAGCT CCCAGCTCCCAGGACTCCGCTGGCTCCTC-3' / 5'-GGCTTAGGATTGTTAC GCCCTCACTTATCTACTAATCAATTA-3'
$Donor_{no\ cut}$ (CMV-EGFP / CMV-EGFP-polyA)	5'-TCCACTGCGACGTCGCGAGTGGTATGCCC CTCGCTAATACTTAGTCACCTACTAGTTAG-3' / 5'-CCGATCTGTTATAGATCGCGGGCTTAGGATT GTTACGCCCTCACTTATCTACTAATCA-3'	

Supplementary Table 2. Primers used for PCR amplification of the targeted genome sequences.

Gene Name	Primer Pairs	Sequence
CSPG4	CSPG4-L/R	5'-CACGGGCCCTTTAAGAAGGT-3' / 5'-GGACCCACTTCTCACTGTCG-3'
LRP1	LRP1-L/R	5'-AGCCTAGAGGACTTGGAGGG-3' / 5'-CAAAGCTGTCAGCCCAGAGA-3'
ANTXR1	sgRNA3-L/R	5'-CGGAACGTCTGAATGCACTG-3' / 5'-ACAGCACACCCCAATCCAG-3'
ANTXR1	sgRNA6-L/R	5'-TGCGTCCTTCACAAGCATCA-3' / 5'-CCATCCTTGCAGTGCTGGTA-3'
ANTXR1	sgRNA10-L/R	5'-TTGCTTCCAGGCCCAAAAGA-3' / 5'-TGCCATCAATAAAGGCAGGGTA-3'
ANTXR1	sgRNA14-L/R	5'-TGTTCTGCGTTAGGAGGACC-3' / 5'-AGAGTGAGCCACTCGGTAGA-3'
ANTXR1	sgRNA16-L/R	5'-ATGGTTGACCCTGAACGGAT-3' / 5'-TGTGGCGTGAATTTGTGACTG-3'
ANTXR1	sgRNA24-L/R	5'-GGAATCCAGGGTTGGGTAGC-3' / 5'-CTGATACACTGGCAGGAGGC-3'
ANTXR1	sgRNA47-L/R	5'-CAGGCCAAGCGTTCATCAAG-3' / 5'-CAGAGTAGCGCCCCTTTCAA-3'
ANTXR1	sgRNA58-L/R	5'-AATAAAGGACCCGCGAGGAA-3' / 5'-GTGCCCGGTGAGAAGTATGC-3'
ANTXR1	sgRNA62-L/R	5'-AACTAGCTGGCAAGTTGGGA-3' / 5'-GGAAACGCACAGTGAAAGGC-3'

Supplementary Table 3. Primers used for PCR amplification of the sgRNA-coding regions integrated into the genomes.

Primer Pairs	Sequence
L/R	5'-TATCTTGTGGAAAGGACGAAACACC-3' / 5'-AATACGGTTATCCACGCGGC-3'

Supplementary data Primers for sgRNA or pgRNA construction.

sgRNA	Targeted sequence (PAM)	Primers (Forward/Reverse)
$sgRNA_{Negative}$	GGCTTAGGATTGTTACGCCC	5'-ACCGGGCTTAGGATTGTTACGCCC-3' / 5'-AAACGGGCGTAACAATCCTAAGCC-3'
$sgRNA1_{Negative}$	TGACGCGATAGAGTTGGCTT	5'-ACCGTGACGCGATAGAGTTGGCTT-3' / 5'-AAACAAGCCAACTCTATCGCGTCA-3'
$sgRNA2_{Negative}$	TTCACCGTCCACGTGCGCAT	5'-ACCGTTCACCGTCCACGTGCGCAT-3' / 5'-AAACATGCGCACGTGGACGGTGAA-3'
$sgRNA3_{Negative}$	TTCTTCGGCCTACACCCGGT	5'-ACCGTTCTTCGGCCTACACCCGGT-3' / 5'-AAACACCGGGTGTAGGCCGAAGAA-3'
$sgRNA4_{Negative}$	GGAATTACGACTAACCGATT	5'-ACCGGGAATTACGACTAACCGATT-3' / 5'-AAACAATCGGTTAGTCGTAATTCC-3'
$sgRNA5_{Negative}$	TGCGGGGAGCTCTCTTAATG	5'-ACCGTGCGGGGAGCTCTCTTAATG-3' / 5'-AAACCATTAAGAGAGCTCCCCGCA-3'

sgRNA6$_{Negative}$	TAGAGATATCCGATCGTGGT	5’-ACCGTAGAGATATCCGATCGTGGT-3’ / 5’-AAACACCACGATCGGATATCTCTA-3’
sgRNA7$_{Negative}$	GGGTGCCCACTAATAGCCGC	5’-ACCGGGGTGCCCACTAATAGCCGC-3’ / 5’-AAACGCGGCTATTAGTGGGCACCC-3’
sgRNA8$_{Negative}$	TGCAGTCGCGCTGAGCGTCA	5’-ACCGTGCAGTCGCGCTGAGCGTCA-3’ / 5’-AAACTGACGCTCAGCGCGACTGCA-3’
sgRNA9$_{Negative}$	GGATTGAATGGCTAACGCGG	5’-ACCGGGATTGAATGGCTAACGCGG-3’ / 5’-AAACCCGCGTTAGCCATTCAATCC-3’
sgRNA10$_{Negative}$	GCGGTTCCGGGAGCACATTT	5’-ACCGGCGGTTCCGGGAGCACATTT-3’ / 5’-AAACAAATGTGCTCCCGGAACCGC-3’
sgRNA11$_{Negative}$	GGCGCATTAAAGTCGAGAGC	5’-ACCGGGCGCATTAAAGTCGAGAGC-3’ / 5’-AAACGCTCTCGACTTTAATGCGCC-3’
sgRNA12$_{Negative}$	GACGTAGCCTTCCGAAATAT	5’-ACCGGACGTAGCCTTCCGAAATAT-3’ / 5’-AAACATATTTCGGAAGGCTACGTC-3’
sgRNA13$_{Negative}$	TGAAGTGGGGCGTCGGACAC	5’-ACCGTGAAGTGGGGCGTCGGACAC-3’ / 5’-AAACGTGTCCGACGCCCCACTTCA-3’
sgRNA14$_{Negative}$	CTATATTGTCGCGCAGTGGA	5’-ACCGCTATATTGTCGCGCAGTGGA-3’ / 5’-AAACTCCACTGCGCGACAATATAG-3’
sgRNA15$_{Negative}$	CGGCTTTGTTGCCCGTAAGC	5’-ACCGCGGCTTTGTTGCCCGTAAGC-3’ / 5’-AAACGCTTACGGGCAACAAAGCCG-3’
sgRNA16$_{Negative}$	TCCCGGTTGGTGAACGATAC	5’-ACCGTCCCGGTTGGTGAACGATAC-3’ / 5’-AAACGTATCGTTCACCAACCGGGA-3’
sgRNA17$_{Negative}$	TTAACTCGAACGCTCGAAAG	5’-ACCGTTAACTCGAACGCTCGAAAG-3’ / 5’-AAACCTTTCGAGCGTTCGAGTTAA-3’
sgRNA18$_{Negative}$	GGACTATCCACCGTTTACTC	5’-ACCGGGACTATCCACCGTTTACTC-3’ / 5’-AAACGAGTAAACGGTGGATAGTCC-3’
sgRNA$_{CSPG4}$	TTGGCCAGACTTGCATCCGCGGG	5’-ACCGTTGGCCAGACTTGCATCCGC-3’ / 5’-AAACGCGGATGCAAGTCTGGCCAA-3’
sgRNA1$_{CSPG4}$	CCCACTTCCAGCCCCCGGCCTGG	5’-ACCGCCCACTTCCAGCCCCCGGCC-3’ / 5’-AAACGGCCGGGGGCTGGAAGTGGG-3’
sgRNA2$_{CSPG4}$	AGTGCGCGGACACCCGGAGCCGG	5’-ACCGAGTGCGCGGACACCCGGAGC-3’ / 5’-AAACGCTCCGGGTGTCCGCGCACT-3’
sgRNA3$_{CSPG4}$	CCACTCAGCTCCCAGCTCCCAGG	5’-ACCGCCACTCAGCTCCCAGCTCCC-3’ / 5’-AAACGGGAGCTGGGAGCTGAGTGG-3’
sgRNA4$_{CSPG4}$	GTCTGCGCCTGCCCCGCCCCCGG	5’-ACCGGTCTGCGCCTGCCCCGCCCC-3’ / 5’-AAACGGGGCGGGGCAGGCGCAGAC-3’
sgRNA5$_{CSPG4}$	TCCAGCCCCCGGCCTGGCCTTGG	5’-ACCGTCCAGCCCCCGGCCTGGCCT-3’ / 5’-AAACAGGCCAGGCCGGGGGCTGGA-3’
sgRNA6$_{CSPG4}$	TTGGCCAGACTTGCATCCGCGGG	5’-ACCGTTGGCCAGACTTGCATCCGC-3’ / 5’-AAACGCGGATGCAAGTCTGGCCAA-3’
sgRNA7$_{CSPG4}$	TGCATCCGCGGGTGAGTGAGGGG	5’-ACCGTGCATCCGCGGGTGAGTGAG-3’ / 5’-AAACCTCACTCACCCGCGGATGCA-3’
sgRNA8$_{CSPG4}$	CTCCGGGAGGGAAAAGGGACAGG	5’-ACCGCTCCGGGAGGGAAAAGGGAC-3’ / 5’-AAACGTCCCTTTTCCCTCCCGGAG-3’
sgRNA9$_{CSPG4}$	GGGACAGGAGGAGCCAGAAGTGG	5’-ACCGGGGACAGGAGGAGCCAGAAG-3’ / 5’-AAACCTTCTGGCTCCTCCTGTCCC-3’
sgRNA10$_{CSPG4}$	GAGTTAAGTGCGCGGACACCCGG	5’-ACCGGAGTTAAGTGCGCGGACACC-3’ / 5’-AAACGGTGTCCGCGCACTTAACTC-3’
sgRNA11$_{CSPG4}$	CCCAGCTCCCAGGACTCCGCTGG	5’-ACCGCCCAGCTCCCAGGACTCCGC-3’ / 5’-AAACGCGGAGTCCTGGGAGCTGGG-3’
sgRNA12$_{CSPG4}$	GTCCTGCCGCCCAGCCCGCCGGG	5’-ACCGGTCCTGCCGCCCAGCCCGCC-3’ / 5’-AAACGGCGGGCTGGGCGGCAGGAC-3’
sgRNA13$_{CSPG4}$	CAGCCCGCCGGGATGCAGTCCGG	5’-ACCGCAGCCCGCCGGGATGCAGTC-3’ / 5’-AAACGACTGCATCCCGGCGGGCTG-3’
sgRNA14$_{CSPG4}$	CCGGGATGCAGTCCGGGCCGCGG	5’-ACCGCCGGGATGCAGTCCGGGCCG-3’ / 5’-AAACCGGCCCGGACTGCATCCCGG-3’
sgRNA15$_{CSPG4}$	GGCTTTGACCCTGACTATGTTGG	5’-ACCGGGCTTTGACCCTGACTATGT-3’ / 5’-AAACACATAGTCAGGGTCAAAGCC-3’
sgRNA16$_{CSPG4}$	CCCCAGATCGGGAGTCAAGCAGG	5’-ACCGCCCCAGATCGGGAGTCAAGC-3’ / 5’-AAACGCTTGACTCCCGATCTGGGG-3’
sgRNA1$_{LRP1}$	ACCGCAGATACACAGTGTCTAGG	5’-ACCGACCGCAGATACACAGTGTCT-3’ / 5’-AAACAGACACTGTGTATCTGCGGT-3’
sgRNA2$_{LRP1}$	AGCCACCCCCTGACACCCCCAGG	5’-ACCGAGCCACCCCCTGACACCCCC-3’ / 5’-AAACGGGGGTGTCAGGGGGTGGCT-3’
sgRNA3$_{LRP1}$	ACCCCCAGGCACATAGACCATGG	5’-ACCGACCCCCAGGCACATAGACCA-3’ / 5’-AAACTGGTCTATGTGCCTGGGGGT-3’

sgRNA4$_{LRP1}$	GGCACATAGACCATGGCTGAGGG	5'-ACCGGGCACATAGACCATGGCTGA-3' / 5'-AAACTCAGCCATGGTCTATGTGCC-3'
sgRNA5$_{LRP1}$	TAGACCATGGCTGAGGGTCTGGG	5'-ACCGTAGACCATGGCTGAGGGTCT-3' / 5'-AAACAGACCCTCAGCCATGGTCTA-3'
sgRNA6$_{LRP1}$	GGCTGAGGGTCTGGCCAGATGG	5'-ACCGGGCTGAGGGTCTGGCCAGA-3' / 5'-AAACTCTGGCCCAGACCCTCAGCC-3'
sgRNA7$_{LRP1}$	GCCAGATGGAGACTGATGCTAGG	5'-ACCGGCCAGATGGAGACTGATGCT-3' / 5'-AAACAGCATCAGTCTCCATCTGGC-3'
sgRNA8$_{LRP1}$	AGGGAAGGAAGAGCAGTACTAGG	5'-ACCGAGGGAAGGAAGAGCAGTACT-3' / 5'-AAACAGTACTGCTCTTCCTTCCCT-3'
sgRNA9$_{LRP1}$	GAGCAGTACTAGGGGACTTTGGG	5'-ACCGGAGCAGTACTAGGGGACTTT-3' / 5'-AAACAAAGTCCCCTAGTACTGCTC-3'
sgRNA10$_{LRP1}$	GGACTTTGGGTTCACAGAGTTGG	5'-ACCGGGACTTTGGGTTCACAGAGT-3' / 5'-AAACACTCTGTGAACCCAAAGTCC-3'
sgRNA11$_{LRP1}$	TTAACTTATCCCTCTTCCTTTGG	5'-ACCGTTAACTTATCCCTCTTCCTT-3' / 5'-AAACAAGGAAGAGGGATAAGTTAA-3'
sgRNA12$_{LRP1}$	TCTTCCTTTGGCAGATGTTTGGG	5'-ACCGTCTTCCTTTGGCAGATGTTT-3' / 5'-AAACAAACATCTGCCAAAGGAAGA-3'
sgRNA13$_{LRP1}$	TTTGGCAGATGTTTGGGAGGAGG	5'-ACCGTTTGGCAGATGTTTGGGAGG-3' / 5'-AAACCCTCCCAAACATCTGCCAAA-3'
sgRNA14$_{LRP1}$	AGCTGATCCCTAGCCTGATGTGG	5'-ACCGAGCTGATCCCTAGCCTGATG-3' / 5'-AAACCATCAGGCTAGGGATCAGCT-3'
sgRNA15$_{LRP1}$	CCCCTTCAAAATGCTTGGCATGG	5'-ACCGCCCCTTCAAAATGCTTGGCA-3' / 5'-AAACTGCCAAGCATTTTGAAGGGG-3'
sgRNA16$_{LRP1}$	TGCTTGGCATGGTGCCTGTTGGG	5'-ACCGTGCTTGGCATGGTGCCTGTT-3' / 5'-AAACAACAGGCACCATGCCAAGCA-3'
sgRNA17$_{LRP1}$	CCAGATGGAGACTGATGCTAGGG	5'-ACCGTCCTTTGGCAGATGTTTGGG-3' / 5'-AAACCCCAAACATCTGCCAAAGGA-3'
sgRNA1$_{ANTXR1}$	TGGAGAGTACCACTTCCGGGGGG	5'-ACCGTGGAGAGTACCACTTCCGGG-3' / 5'-AAACCCCGGAAGTGGTACTCTCCA-3'
sgRNA2$_{ANTXR1}$	CTTCCGACATGCCCGCAACGTGG	5'-ACCGCTTCCGACATGCCCGCAACG-3' / 5'-AAACCGTTGCGGGCATGTCGGAAG-3'
sgRNA3$_{ANTXR1}$	ATGACTTACACAGTGTGTGGTGG	5'-ACCGATGACTTACACAGTGTGTGG-3' / 5'-AAACCCACACACTGTGTAAGTCAT-3'
sgRNA4$_{ANTXR1}$	CTCCAGGTCAGCATGAACGATGG	5'-ACCGCTCCAGGTCAGCATGAACGA-3' / 5'-AAACTCGTTCATGCTGACCTGGAG-3'
sgRNA5$_{ANTXR1}$	TTGGAGAGTACCACTTCCGGGGG	5'-ACCGTTGGAGAGTACCACTTCCGG-3' / 5'-AAACCCGGAAGTGGTACTCTCCAA-3'
sgRNA6$_{ANTXR1}$	TTTCCTCAGTAGGACCCACAAGG	5'-ACCGTTTCCTCAGTAGGACCCACA-3' / 5'-AAACTGTGGGTCCTACTGAGGAAA-3'
sgRNA7$_{ANTXR1}$	ACATGCCCGCAACGTGGACAGGG	5'-ACCGACATGCCCGCAACGTGGACA-3' / 5'-AAACTGTCCACGTTGCGGGCATGT-3'
sgRNA8$_{ANTXR1}$	ATCTCAACAACAATATGCGTCGG	5'-ACCGATCTCAACAACAATATGCGT-3' / 5'-AAACACGCATATTGTTGTTGAGAT-3'
sgRNA9$_{ANTXR1}$	GTAGACGCCTCTTATTATGGTGG	5'-ACCGGTAGACGCCTCTTATTATGG-3' / 5'-AAACCCATAATAAGAGGCGTCTAC-3'
sgRNA10$_{ANTXR1}$	AGTCAAAGCAATGATGACGCTGG	5'-ACCGAGTCAAAGCAATGATGACGC-3' / 5'-AAACGCGTCATCATTGCTTTGACT-3'
sgRNA11$_{ANTXR1}$	ATTGGAGAGTACCACTTCCGGGG	5'-ACCGATTGGAGAGTACCACTTCCG-3' / 5'-AAACCGGAAGTGGTACTCTCCAAT-3'
sgRNA12$_{ANTXR1}$	TAGACGCCTCTTATTATGGTGGG	5'-ACCGTAGACGCCTCTTATTATGGT-3' / 5'-AAACACCATAATAAGAGGCGTCTA-3'
sgRNA13$_{ANTXR1}$	TTTATTGTTTTCTCCACCCGAGG	5'-ACCGTTTATTGTTTTCTCCACCCG-3' / 5'-AAACCGGGTGGAGAAAACAATAAA-3'
sgRNA14$_{ANTXR1}$	TCATTTCAAGTTGTCGTGAGAGG	5'-ACCGTCATTTCAAGTTGTCGTGAG-3' / 5'-AAACCTCACGACAACTTGAAATGA-3'
sgRNA15$_{ANTXR1}$	TGTCCACGTTGCGGGCATGTCGG	5'-ACCGTGTCCACGTTGCGGGCATGT-3' / 5'-AAACACATGCCCGCAACGTGGACA-3'
sgRNA16$_{ANTXR1}$	TTCAGAGAACAAATCCGTCAAGG	5'-ACCGTTCAGAGAACAAATCCGTCA-3' / 5'-AAACTGACGGATTTGTTCTCTGAA-3'
sgRNA17$_{ANTXR1}$	TAATAAGAGGCGTCTACCGTTGG	5'-ACCGTAATAAGAGGCGTCTACCGT-3' / 5'-AAACACGGTAGACGCCTCTTATTA-3'
sgRNA18$_{ANTXR1}$	ATGCCTTGTGGGTCCTACTGAGG	5'-ACCGATGCCTTGTGGGTCCTACTG-3' / 5'-AAACCAGTAGGACCCACAAGGCAT-3'
sgRNA19$_{ANTXR1}$	AACTTTGGGCTCTAGACAGAAGG	5'-ACCGAACTTTGGGCTCTAGACAGA-3' / 5'-AAACTCTGTCTAGAGCCCAAAGTT-3'

sgRNA20$_{ANTXR1}$	GAAAGATTTCAATGAGACACAGG	5’-ACCGGAAAGATTTCAATGAGACAC-3’ / 5’-AAACGTGTCTCATTGAAATCTTTC-3’
sgRNA21$_{ANTXR1}$	GAGGGCTCTCCGCTCCGCCGTGG	5’-ACCGGAGGGCTCTCCGCTCCGCCG-3’ / 5’-AAACCGGCGGAGCGGAGAGCCCTC-3’
sgRNA22$_{ANTXR1}$	GTGTGGTAGGCGTTGTTGAGTGG	5’-ACCGGTGTGGTAGGCGTTGTTGAG-3’ / 5’-AAACCTCAACAACGCCTACCACAC-3’
sgRNA23$_{ANTXR1}$	TTGTGGGTCCTACTGAGGAAAGG	5’-ACCGTTGTGGGTCCTACTGAGGAA-3’ / 5’-AAACTTCCTCAGTAGGACCCACAA-3’
sgRNA24$_{ANTXR1}$	TGCGTCGGCCTTCTTCCCCCCGG	5’-ACCGTGCGTCGGCCTTCTTCCCCC-3’ / 5’-AAACGGGGGAAGAAGGCCGACGCA-3’
sgRNA25$_{ANTXR1}$	GAAGTGGTACTCTCCAATCAAGG	5’-ACCGGAAGTGGTACTCTCCAATCA-3’ / 5’-AAACTGATTGGAGAGTACCACTTC-3’
sgRNA26$_{ANTXR1}$	GAACCATCCACCATATGTGCAGG	5’-ACCGGAACCATCCACCATATGTGC-3’ / 5’-AAACGCACATATGGTGGATGGTTC-3’
sgRNA27$_{ANTXR1}$	ATCTCGAGACCTATTAGCCTGGG	5’-ACCGATCTCGAGACCTATTAGCCT-3’ / 5’-AAACAGGCTAATAGGTCTCGAGAT-3’
sgRNA28$_{ANTXR1}$	TCTGTGATGCGTCCACAGCCAGG	5’-ACCGTCTGTGATGCGTCCACAGCC-3’ / 5’-AAACGGCTGTGGACGCATCACAGA-3’
sgRNA29$_{ANTXR1}$	TGTTGAGATTTCGCGGCTCAGGG	5’-ACCGTGTTGAGATTTCGCGGCTCA-3’ / 5’-AAACTGAGCCGCGAAATCTCAACA-3’
sgRNA30$_{ANTXR1}$	CATTAAGGTTGTTCCTCGGGTGG	5’-ACCGCATTAAGGTTGTTCCTCGGG-3’ / 5’-AAACCCCGAGGAACAACCTTAATG-3’
sgRNA31$_{ANTXR1}$	TATTGTGTTAAACAGGGTACAGG	5’-ACCGTATTGTGTTAAACAGGGTAC-3’ / 5’-AAACGTACCCTGTTTAACACAATA-3’
sgRNA32$_{ANTXR1}$	ACGAGTCCTACCGTGTCTCCTGG	5’-ACCGACGAGTCCTACCGTGTCTCC-3’ / 5’-AAACGGAGACACGGTAGGACTCGT-3’
sgRNA33$_{ANTXR1}$	TGTGGTAGGCGTTGTTGAGTGGG	5’-ACCGTGTGGTAGGCGTTGTTGAGT-3’ / 5’-AAACACTCAACAACGCCTACCACA-3’
sgRNA34$_{ANTXR1}$	AGAGGACCCTGTCCACGTTGCGG	5’-ACCGAGAGGACCCTGTCCACGTTG-3’ / 5’-AAACCAACGTGGACAGGGTCCTCT-3’
sgRNA35$_{ANTXR1}$	GGGCCATGGCCACGGCGGAGCGG	5’-ACCGGGGCCATGGCCACGGCGGAG-3’ / 5’-AAACCTCCGCCGTGGCCATGGCCC-3’
sgRNA36$_{ANTXR1}$	TTACCTCACTCTCCTCGGCAGGG	5’-ACCGTTACCTCACTCTCCTCGGCA-3’ / 5’-AAACTGCCGAGGAGAGTGAGGTAA-3’
sgRNA37$_{ANTXR1}$	ATATTGTTGTTGAGATTTCGCGG	5’-ACCGATATTGTTGTTGAGATTTCG-3’ / 5’-AAACCGAAATCTCAACAACAATAT-3’
sgRNA38$_{ANTXR1}$	CCCTCGGCATCGGCTTCCAGTGG	5’-ACCGCCCTCGGCATCGGCTTCCAG-3’ / 5’-AAACCTGGAAGCCGATGCCGAGGG-3’
sgRNA39$_{ANTXR1}$	CAGGTCAAATCCGCCGTAGCAGG	5’-ACCGCAGGTCAAATCCGCCGTAGC-3’ / 5’-AAACGCTACGGCGGATTTGACCTG-3’
sgRNA40$_{ANTXR1}$	GCTCATCTGCGCCGGGCAAGGGG	5’-ACCGGCTCATCTGCGCCGGGCAAG-3’ / 5’-AAACCTTGCCCGGCGCAGATGAGC-3’
sgRNA41$_{ANTXR1}$	GGGAAAACTCGATGCCTTGTGGG	5’-ACCGGGGAAAACTCGATGCCTTGT-3’ / 5’-AAACACAAGGCATCGAGTTTTCCC-3’
sgRNA42$_{ANTXR1}$	TCAAATCCGCCGTAGCAGGCTGG	5’-ACCGTCAAATCCGCCGTAGCAGGC-3’ / 5’-AAACGCCTGCTACGGCGGATTTGA-3’
sgRNA43$_{ANTXR1}$	GGCCATCGTTCATGCTGACCTGG	5’-ACCGGGCCATCGTTCATGCTGACC-3’ / 5’-AAACGGTCAGCATGAACGATGGCC-3’
sgRNA44$_{ANTXR1}$	GGGGGTCCAGCCTGCTACGGCGG	5’-ACCGGGGGGTCCAGCCTGCTACGG-3’ / 5’-AAACCCGTAGCAGGCTGGACCCCC-3’
sgRNA45$_{ANTXR1}$	GGCCCGGATTGCGGACAGTAAGG	5’-ACCGGGCCCGGATTGCGGACAGTA-3’ / 5’-AAACTACTGTCCGCAATCCGGGCC-3’
sgRNA46$_{ANTXR1}$	ACTCTGGTGCTCATCTGCGCCGG	5’-ACCGACTCTGGTGCTCATCTGCGC-3’ / 5’-AAACGCGCAGATGAGCACCAGAGT-3’
sgRNA47$_{ANTXR1}$	GACATGCCCGCAACGTGGACAGG	5’-ACCGGACATGCCCGCAACGTGGAC-3’ / 5’-AAACGTCCACGTTGCGGGCATGTC-3’
sgRNA48$_{ANTXR1}$	GATCCTTACTGTCCGCAATCCGG	5’-ACCGGATCCTTACTGTCCGCAATC-3’ / 5’-AAACGATTGCGGACAGTAAGGATC-3’
sgRNA49$_{ANTXR1}$	GATTGGAGAGTACCACTTCCGGG	5’-ACCGGATTGGAGAGTACCACTTCC-3’ / 5’-AAACGGAAGTGGTACTCTCCAATC-3’
sgRNA50$_{ANTXR1}$	TTGTTGAGTGGGTACTTGGCTGG	5’-ACCGTTGTTGAGTGGGTACTTGGC-3’ / 5’-AAACGCCAAGTACCCACTCAACAA-3’
sgRNA51$_{ANTXR1}$	GAGGTGGGGGAGTGTAGATGGGG	5’-ACCGGAGGTGGGGGAGTGTAGATG-3’ / 5’-AAACCATCTACACTCCCCCACCTC-3’
sgRNA52$_{ANTXR1}$	CAGGAAGTGTGCTGCACCACTGG	5’-ACCGCAGGAAGTGTGCTGCACCAC-3’ / 5’-AAACGTGGTGCAGCACACTTCCTG-3’

sgRNA53$_{ANTXR1}$	TGATTGGAGAGTACCACTTCCGG	5’-ACCGTGATTGGAGAGTACCACTTC-3’ / 5’-AAACGAAGTGGTACTCTCCAATCA-3’
sgRNA54$_{ANTXR1}$	ATCCTTACTGTCCGCAATCCGGG	5’-ACCGATCCTTACTGTCCGCAATCC-3’ / 5’-AAACGGATTGCGGACAGTAAGGAT-3’
sgRNA55$_{ANTXR1}$	GGGGACGGGATGGGAGGGGTAGG	5’-ACCGGGGGACGGGATGGGAGGGGT-3’ / 5’-AAACACCCCTCCCATCCCGTCCCC-3’
sgRNA56$_{ANTXR1}$	CGGGCAAGGGGGACGCAGGGAGG	5’-ACCGCGGGCAAGGGGGACGCAGGG-3’ / 5’-AAACCCCTGCGTCCCCCTTGCCCG-3’
sgRNA57$_{ANTXR1}$	AGCCTGAAAGCCGTCATTCACGG	5’-ACCGAGCCTGAAAGCCGTCATTCA-3’ / 5’-AAACTGAATGACGGCTTTCAGGCT-3’
sgRNA58$_{ANTXR1}$	TGAGTGGGTACTTGGCTGGCTGG	5’-ACCGTGAGTGGGTACTTGGCTGGC-3’ / 5’-AAACGCCAGCCAAGTACCCACTCA-3’
sgRNA59$_{ANTXR1}$	CTCTGGTGCTCATCTGCGCCGGG	5’-ACCGCTCTGGTGCTCATCTGCGCC-3’ / 5’-AAACGGCGCAGATGAGCACCAGAG-3’
sgRNA60$_{ANTXR1}$	TATTATGGTGGGAGAGGCGTTGG	5’-ACCGTATTATGGTGGGAGAGGCGT-3’ / 5’-AAACACGCCTCTCCCACCATAATA-3’
sgRNA61$_{ANTXR1}$	TCACTCTCCTCGGCAGGGGGTGG	5’-ACCGTCACTCTCCTCGGCAGGGGG-3’ / 5’-AAACCCCCCTGCCGAGGAGAGTGA-3’
sgRNA62$_{ANTXR1}$	GGGGGAGTGTAGATGGGGGCAGG	5’-ACCGGGGGGAGTGTAGATGGGGGC-3’ / 5’-AAACGCCCCCATCTACACTCCCCC-3’
sgRNA63$_{ANTXR1}$	AGAACCACCAGAGGAGAGCCAGG	5’-ACCGAGAACCACCAGAGGAGAGCC-3’ / 5’-AAACGGCTCTCCTCTGGTGGTTCT-3’
sgRNA64$_{ANTXR1}$	GCGCCGGGCAAGGGGGACGCAGG	5’-ACCGGCGCCGGGCAAGGGGGACGC-3’ / 5’-AAACGCGTCCCCCTTGCCCGGCGC-3’
sgRNA65$_{ANTXR1}$	TGGGAGGGGTAGGGGCGCTGGGG	5’-ACCGTGGGAGGGGTAGGGGCGCTG-3’ / 5’-AAACCAGCGCCCCTACCCCTCCCA-3’
sgRNA66$_{ANTXR1}$	GGAGGAGGTGCCCTGTTGGGAGG	5’-ACCGGGAGGAGGTGCCCTGTTGGG-3’ / 5’-AAACCCCAACAGGGCACCTCCTCC-3’
sgRNA67$_{ANTXR1}$	GGGAGGGGTAGGGGCGCTGGGGG	5’-ACCGGGGAGGGGTAGGGGCGCTGG-3’ / 5’-AAACCCAGCGCCCCTACCCCTCCC-3’
sgRNA68$_{ANTXR1}$	GGCAGTGGGGCGCAGGAGGTGGG	5’-ACCGGGCAGTGGGGCGCAGGAGGT-3’ / 5’-AAACACCTCCTGCGCCCCACTGCC-3’
sgRNA69$_{ANTXR1}$	TTGTTGAGATTTCGCGGCTCAGG	5’-ACCGTTGTTGAGATTTCGCGGCTC-3’ / 5’-AAACGAGCCGCGAAATCTCAACAA-3’
sgRNA70$_{ANTXR1}$	ATGGGAGGGGTAGGGGCGCTGGG	5’-ACCGATGGGAGGGGTAGGGGCGCT-3’ / 5’-AAACAGCGCCCCTACCCCTCCCAT-3’
sgRNA71$_{ANTXR1}$	GTAAAGAGACACACCTTGATTGG	5’-ACCGGTAAAGAGACACACCTTGAT-3’ / 5’-AAACATCAAGGTGTGTCTCTTTAC-3’
sgRNA72$_{ANTXR1}$	TGCTCATCTGCGCCGGGCAAGGG	5’-ACCGTGCTCATCTGCGCCGGGCAA-3’ / 5’-AAACTTGCCCGGCGCAGATGAGCA-3’
sgRNA73$_{ANTXR1}$	GTGCTCATCTGCGCCGGGCAAGG	5’-ACCGGTGCTCATCTGCGCCGGGCA-3’ / 5’-AAACTGCCCGGCGCAGATGAGCAC-3’
sgRNA74$_{ANTXR1}$	ACGGTAGACGCCTCTTATTATGG	5’-ACCGACGGTAGACGCCTCTTATTA-3’ / 5’-AAACTAATAAGAGGCGTCTACCGT-3’
sgRNA75$_{ANTXR1}$	TTGTTTATTTTAGGTTCGTTGGG	5’-ACCGTTGTTTATTTTAGGTTCGTT-3’ / 5’-AAACAACGAACCTAAAATAAACAA-3’
sgRNA76$_{ANTXR1}$	GGGCAGTGGGGCGCAGGAGGTGG	5’-ACCGGGGCAGTGGGGCGCAGGAGG-3’ / 5’-AAACCCTCCTGCGCCCCACTGCCC-3’
sgRNA77$_{ANTXR1}$	GATGGGAGGGGTAGGGGCGCTGG	5’-ACCGGATGGGAGGGGTAGGGGCGC-3’ / 5’-AAACGCGCCCCTACCCCTCCCATC-3’
